# Effects of daily static stretch training over 6 weeks on maximal strength, muscle thickness, contraction properties, and flexibility

**DOI:** 10.3389/fspor.2023.1139065

**Published:** 2023-04-17

**Authors:** Tim Wohlann, Konstantin Warneke, Martin Hillebrecht, Astrid Petersmann, Alexander Ferrauti, Stephan Schiemann

**Affiliations:** ^1^ Institute for Exercise, Sport and Health, Leuphana University, Lüneburg, Germany; ^2^ University Sports Centre, Carl von University of Oldenburg, Oldenburg, Germany; ^3^ University Institute for Clinical Chemistry and Laboratory Medicine, University Medicine Oldenburg, Oldenburg, Germany; ^4^ Institute for Clinical Chemistry and Laboratory Medicine, University Medicine Greifswald, Greifswald, Germany; ^5^ Faculty of Sports Science, Ruhr University Bochum, Bochum, Germany

**Keywords:** static stretching, maximal strength, hypertrophy, muscle damage, contraction time, muscle stiffness

## Abstract

**Purpose:**

Static stretch training (SST) with long stretching durations seems to be sufficient to increase flexibility, maximum strength (MSt) and muscle thickness (MTh). However, changes in contraction properties and effects on muscle damage remain unclear. Consequently, the objective of the study was to investigate the effects of a 6-week self-performed SST on MSt, MTh, contractile properties, flexibility, and acute response of creatine kinase (CK) 3 days after SST.

**Methods:**

Forty-four participants were divided into a control (CG, *n* = 22) and an intervention group (IG, *n* = 22), who performed a daily SST for 5 min for the lower limb muscle group. While isometric MSt was measured in leg press, MTh was examined *via* sonography and flexibility by functional tests. Muscle stiffness and contraction time were measured by tensiomyography on the rectus femoris. Additionally, capillary blood samples were taken in the pretest and in the first 3 days after starting SST to measure CK.

**Results:**

A significant increase was found for MSt (*p* < 0.001, *η*^2^ = 0.195) and flexibility in all functional tests (*p* < 0.001, *η*^2^ > 0.310). Scheffé *post hoc* test did not show significant differences between the rectus femoris muscle inter- and intragroup comparisons for MTh nor for muscle stiffness and contraction time (*p* > 0.05, *η*^2^ < 0.100). Moreover, CK was not significantly different between IG and CG with *p* > 0.05, *η*^2^ = 0.032.

**Discussion:**

In conclusion, the increase in MSt cannot be exclusively explained by muscular hypertrophy or the increased CK-related repair mechanism after acute stretching. Rather, neuronal adaptations have to be considered. Furthermore, daily 5-min SST over 6 weeks does not seem sufficient to change muscle stiffness or contraction time. Increases in flexibility tests could be attributed to a stretch-induced change in the muscle–tendon complex.

## Introduction

In animal studies, chronic static stretch training (SST) performed for 30 min up to 24 h for more than 4 weeks induced significant increases of up to 95% in maximal strength (MSt) (1) and muscle mass of up to 318% (2, 3). In humans, it is well established that SST leads to improved flexibility, but the literature shows conflicting results regarding MSt. While some authors measured long-term increases of +16.8% (*p* < 0.001) in the plantar flexors (4) and +32.4% in the knee extensors (*p* < 0.001) (5) following several weeks of stretching, others were not able to find significant increases (6, 7). There are different explanatory approaches for enhanced stretch-induced MSt, such as morphological, physiological, or neural adaptation (4, 8). Smith et al. (9) showed that SST has the potential to produce high mechanical stress that can cause microtraumatization in the muscle, leading to an increase in creatine kinase (CK) (*p* < 0.05). It is hypothesized that the subsequent repair mechanisms are an anabolic stimulus that contributes to increased MSt by resulting in muscle hypertrophy (10).

However, the current literature shows varied results on muscle thickness (MTh) following SST, ranging from 0% (7, 11) to +15.3% (4). These conflicting results could be attributed to high heterogeneity in study design regarding stretching intensity and stretching duration. The training volume ranges from 4 × 30 s, three times per week (12, 13) to a daily 60 min stretch training for 6 weeks (4). Nevertheless, since MSt increases were found after stretching interventions without improved MTh (14), enhanced MSt after SST cannot be exclusively explained by a hypertrophy effect. There is still insufficient knowledge about further physiological and functional adaptions due to SST over several weeks in humans, such as contraction velocity and muscle stiffness. Apart from stretch-induced MSt increases in animal research, Alway (1) showed a significantly reduced contraction velocity, which was accompanied by a shift in fiber contribution to slower myosin heavy chains in animals. The question arises whether the effects of daily stretching on muscle–tendon structures are transferable to humans since—to the best knowledge of the authors—no previous studies have addressed the long-term effects of daily stretching on contraction velocity in humans.

Currently, little is known about the change in muscle stiffness following several weeks of SST. While there is evidence of decreased muscle stiffness (15, 16), other studies found no changes in muscle stiffness (17, 18). This may be explainable by different study designs. Literature is lacking on whether long-term SST alters muscle stiffness when the goal of SST is to increase MSt. Studies that measure stretch-induced increases in MSt due to SST often examined single muscles only, e.g., the plantar flexors, or used a stretching device (4, 12–14). However, in most activities in daily life and sports, several muscle groups are involved in one complex movement. Therefore, the practical relevance of studies investigating single-joint muscles seems limited. To increase the practical applicability of results, the question arises whether and to which extent self-performed SST of multiple muscles can increase MSt in complex movements involving multiple muscles.

To improve understanding of stretch-induced adaptations of the muscle, it is hypothesized that 6-week self-performed SST using daily 5-min stretching per muscle in four different exercises will increase MSt, MTh, and flexibility. Further hypotheses are that SST will lead to muscle microtraumatization, which can be assessed by increases in CK values following the first 3 days of SST, and that 6 weeks of SST will lead to a decrease in contraction velocity and lower muscle stiffness.

## Materials and methods

To test the hypotheses, participants underwent a daily static stretching routine in a pre- and post-test design. First, ultrasound images of the rectus femoris were obtained, followed by an examination of contractile properties using tensiomyography (TMG). Subsequently, flexibility tests were performed. A small amount of capillary blood was drawn from the subjects for CK measurements. Capillary blood collection and CK measurements were performed at pretest and 24, 48, and 72 h after the start of the intervention. A warm-up set was followed by MSt measurements.

### Participants

Based on previous studies performed by Kokkonen et al. (5) and Nelson et al. (13), a high effect size of *d* = 0.8 can be assumed. Ad hoc sample size calculations using G-Power showed a minimum sample size of 27 with an effect size of *f* = 0.4. A total of 44 active participants were recruited from the university sports center and physical education classes of the university and assigned to an intervention group (IG) or a control group (CG), Participants characteristics are shown in Table 1. Participants stating chronic pain in the lower extremity, injury, or surgery during the last 6 months as well as regular stretching routines were excluded from the study. Participants who were used to regular stretch training and were classified as active if they participated in or performed physical activities such as running, trained at the gym at least twice per week, or joined any university sports. All participants were informed about the procedure and purpose of the present study at the pretest meeting and gave their informed consent. The study was performed in accordance with the Declaration of Helsinki and approved by the Oldenburg Medical Ethics Committee 2021-089.

**Table 1:** Characteristics of participants.

Group	*N* (male/female)	Age	Height (cm)	Body weight (kg)
Intervention group	22 (13/9)	24.2 ± 2.9	183.2 ± 10,1	76.3 ± 12.7
Control group	22 (10/12)	24.8 ± 3.1	174.3 ± 8.5	70.1 ± 16.3

### Measuring maximal isometric strength

Before testing MSt, a warm-up program was performed with running for 5 min, followed by 10 deep bodyweight squats. Isometric MSt was measured unilaterally for both legs against an immovable platform from AST (model KAC) with an integrated strain gauge using a 13-bit AD converter (NI6009) with a measuring range of 5000 N. Participants were positioned on their backs with a hip angle of 80°, a knee angle of 70°, an ankle angle of 90° and performed as many trials until no further increase in MSt values could be obtained. They completed at least three trials with a 90-s rest between trials to avoid fatigue. To counteract a habituation effect, an appointment before the pretest was given where the subjects could practice the MSt tests.

### Measuring range of motion

#### Knee joint

**Figure 1:**
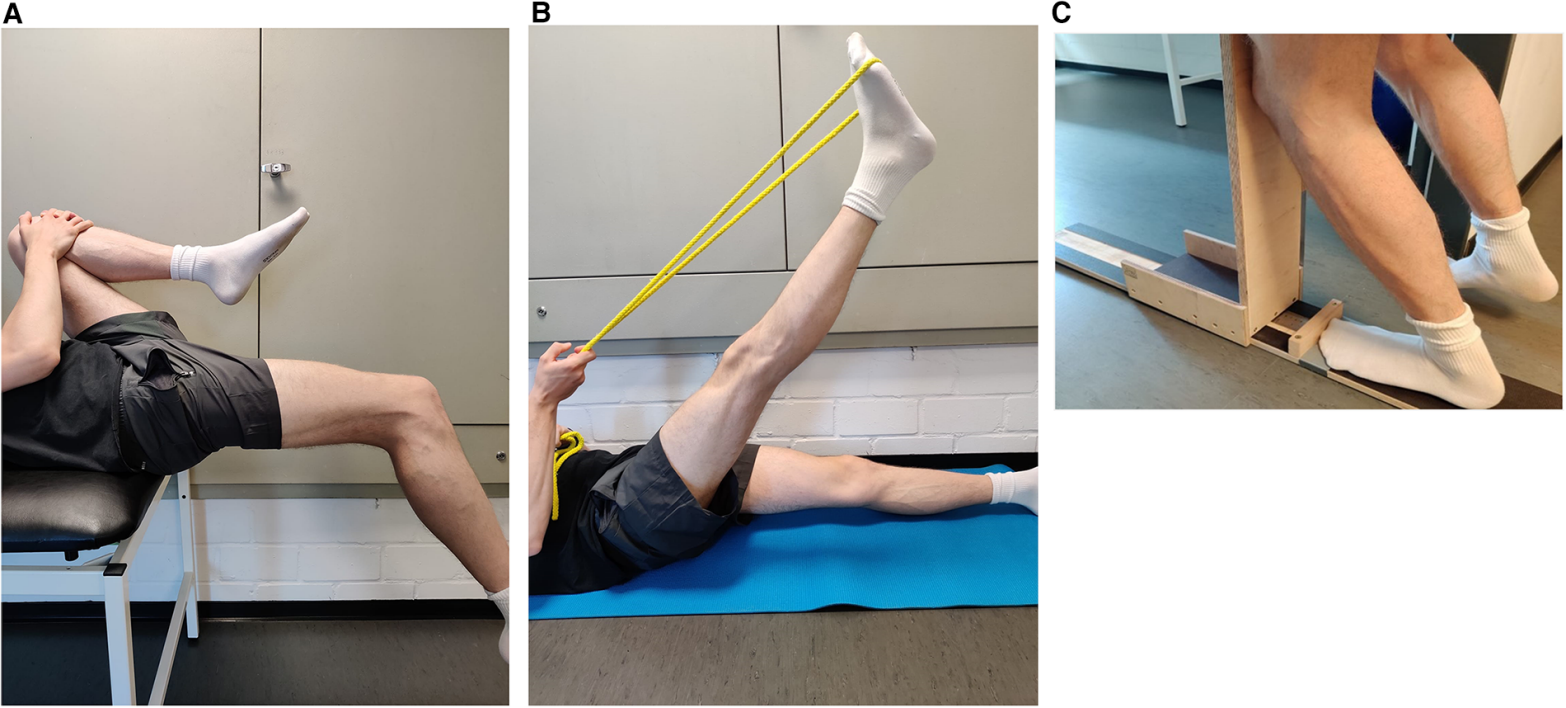
Flexibility tests of the knee joint (**A**), hamstrings muscles (**B**), and ankle joint (**C**).

The flexibility of the knee joint was tested using the modified Thomas test. This test has a high test–retest reliability, with a reported intraclass correlation coefficient (ICC) value of 0.87–0.91 (19). For this purpose, the subjects lay down with their coccyx on the edge of a medical bed so that the legs were not on the medical bed. The non-measured leg was then bent with the hands on the knee toward the umbilicus until the measured leg was parallel to the floor and the angle between the two legs did not change due to a seesaw motion (Figure 1A). After reaching this position, a digital goniometer was used to measure the angle between the lower leg and the unbent upper leg. The goniometer was held at the knee joint and was in line with the thigh (parallel to the ground) and the lateral malleolus.

#### Hamstrings

The hamstring flexibility test was performed as described by Cejudo et al. (19). Subjects lay flat on the floor and raised one leg fully extended as high as possible. The other leg was placed fully extended on the floor (Figure 1B). A digital goniometer was used to measure the angle at the hip between the raised leg and the non-raised leg on the floor. The reliability of this test can be classified as high, with an ICC value of 0.87–0.94 (19).

#### Ankle joint

A knee-to-wall test consisting of a track motion sled was used to determine flexibility in the ankle joint. The measured leg was positioned on the board, and a piece of paper was placed underneath the heel; participants were instructed to bend the knee and push the sled forward until the paper pulled away from the heel. Subsequently, the centimeters were read off the measuring scale (Figure 1C). The reliability of this test is considered high, with ICC ranging between 0.979 and 0.992 and CV ranging between 0.94 and 1.81 (4).

### Measuring muscle thickness

**Figure 2:**
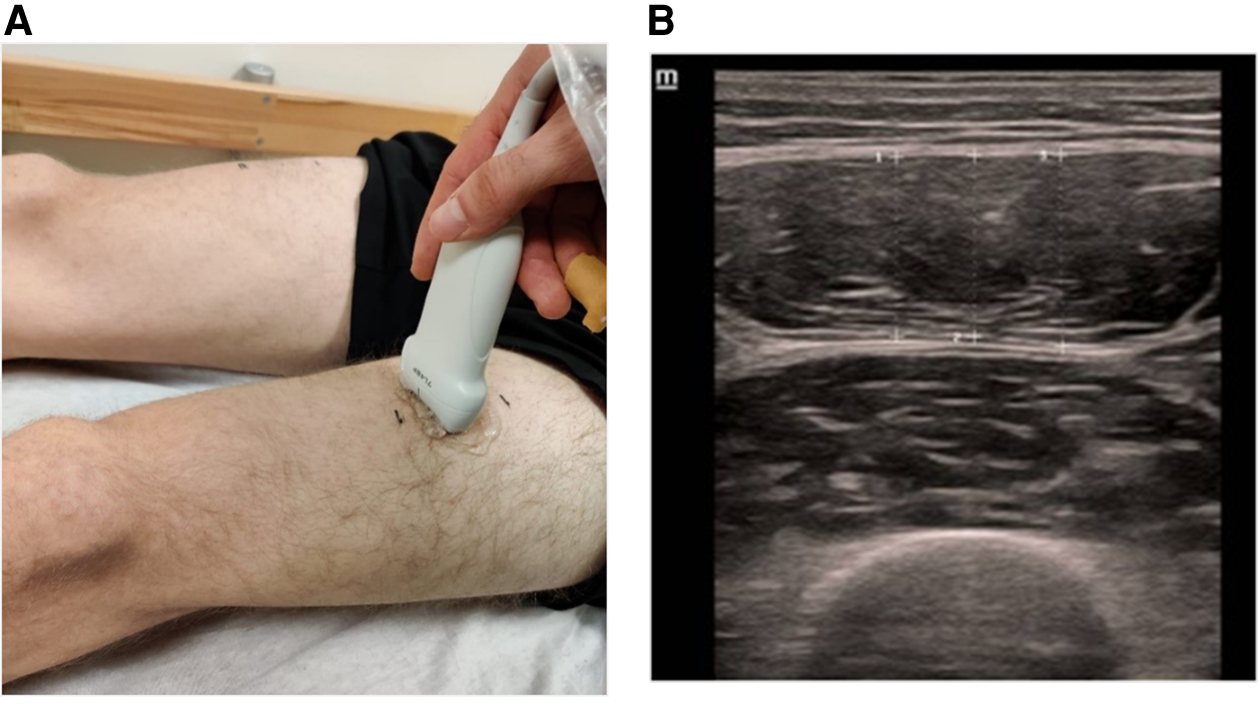
Sonography to measure muscle thickness of m. rectus femoris (**A**) and image analysing of three subsequent distances (**B**).

To measure MTh changes, ultrasound images of the rectus femoris muscle were acquired using a DC 30 Full HD device from MINDRAY with a 5–14 MHz linear probe. A point 15 cm above the superior patella in the direction of the spina iliaca anterior inferior was marked with a waterproof felt-tip pen. This measurement method is described by e Lima et al. (20) with a high-reliability ICC value of 0.88–0.99. For the measurement of MTh, the transducer was positioned in the middle of the muscle belly in a horizontal line orthogonal to the leg (Figure 2A). Three images of the rectus femoris muscle were acquired per leg and test day, each with three subsequent distance measurements centered in the image. Two reliability values were calculated for the present study: First, reliability of the measured distances within 1 image (Figure 2B) and second the average distances between 3 images (Table 2).

**Table 2:** Reliability of the methods used.

Parameter	ICC (95% CI)	CV (in %)	SD
Maximal strength	0.978–0.987	1.8–2.0	11.5–13.9
Muscle thickness within one image	0.982–0.987	1.2–1.3	1.8–2.1
Muscle thickness between three images	0.951–0.971	1.7–2.2	2.6–3.5
Flexibility knee joint	0.964–0.984	1.2–1.3	1.3–1.5
Flexibility hamstrings	0.956–0.966	1.5–1.7	1.1–1.3
Flexibility ankle joint	0.978–0.989	2.3–2.7	0.4–0.5

Within one image, best and second-best value within a measurement; between three images, average best and average second-best value from all three measurements; ICC, intraclass correlation coefficient; CV, coefficient of variance; SD, standard deviation.

### Measuring contractile properties

**Figure 3:**
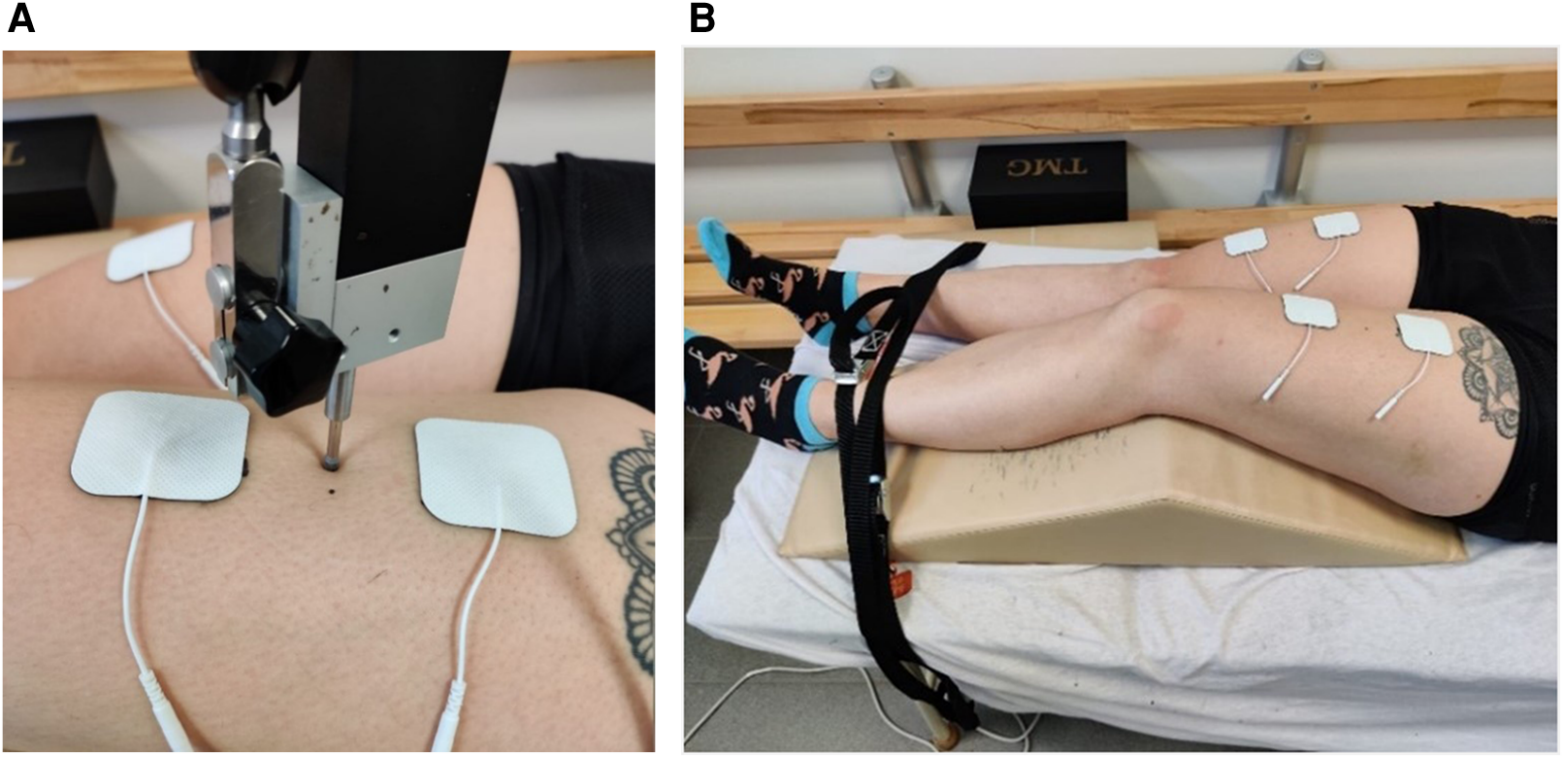
Measuring point of the TMG sensor position (**A**) and setup of the TMG measurement (**B**).

TMG was used to examine contractile properties. The measurement point of the TMG was placed on the same spot as the measurement point of the sonography. Before the electrodes were positioned, the skin was shaved, disinfected with alcohol, and dried. Afterward, the electrodes were placed at an interelectrode distance of 6 cm in a longitudinal direction along the leg, as recommended by Wilson et al. (21) (Figure 3). Two straps were used to fix the leg to avoid leg movements in response to the electric impulse. Electric stimulation started with 60 mA and successively increased by 10 mA until the tmg curve did not change in three consecutive trials. This procedure was used in pre- and post-tests. Parameters were calculated based on the maximal radial displacement curve over time. First, muscle displacement (Dm) expressed in millimetres represents the maximal radial displacement and provides information about the stiffness of the muscle (Simunič et al. (22)). Reliability for rectus femoris was shown by Paula Simola et al. with ICC: 0.92; CV: 9.30% and ICC: 0.92; CV: 5.7% (Wilson et al. (21)). Second, contraction time (Tc) measured in milliseconds provides information about the velocity of muscle contraction and is calculated by the deformation time between 10% and 90% of DM (Paula Simola et al. (24)). Reliability for rectus femoris was shown by Paula Simola et al. (23) with ICC: 0.86; CV: 4.90% and Wilson et al. (21) with CV: 2.0%.

### Measuring creatine kinase

CK was measured before and 24, 48, and 72 h after the intervention started. In total, 150 mL of blood was collected from the fingertip using a sterile disposable device and an EDTA-coated capillary. Samples were transferred to a tube immediately after collection and centrifuged. The plasma supernatant, approximately 70 mL, was removed and used to determine CK activity. The analysis was performed on a CobasPro (Roche Diagnostics Deutschland GmbH, Mannheim, Germany). A photometric assay in which the activity of CK was inferred by measuring NADPH in a defined reaction mixture was conducted.

### Intervention

The intervention group performed a standardized 6-week SST mainly targeting m. quadriceps—rectus femoris, m. gastrocnemius, hamstrings, and m. gluteus maximus for the dominant leg (preferred side for kicking a ball). Each stretch exercise was performed continuously for 5 min, resulting in a daily stretch time of 20 min. The intensity was regulated using a subjective visual analog scale (VAS) ranging from 1 to 10, with 10 defined as the maximal stretch pain. Participants were instructed to perform each stretch exercise at maximum tolerated stretch pain and protocolled each training session. A supervised group stretch training session was offered 3 days per week to improve compliance. It was mandatory to participate in at least one group training session per week.

### Data analysis

Statistical analysis was performed using SPSS 28 (IBM SPSS Statistics, version 28). All data are provided as mean ± standard deviation. Normal distribution was approved by using the Shapiro–Wilk test (*p* > 0.05). For reliability, the intraclass correlation coefficient and the coefficient of variance were calculated (Table 2). One-way analysis of variance (ANOVA) was used to ensure the absence of significant differences in pretest values. Two-way ANOVA with repeated measurements of factor time and group with a Scheffé *post hoc* test was performed to reveal significant differences within and between groups. Significant differences were tested bilaterally. Effect sizes [eta square (*η*^2^)] were categorized as small effect *η*^2 ^< 0.06, medium effect *η*^2 ^= 0.06–0.14, and high effect *η*^2^ > 0.14, as well as Cohen's *d* with *d* < 0.5 indicating small effect, 0.5–0.8 indicating medium effect, and  >0.8 indicating high effect (Cohen, 25). The critical level of significance was set at *p* = 0.05 in this study.

## Results

One-way ANOVA showed no significant differences between pretest values for all parameters (*p* > 0.05). In Tables 3–5, legs are listed as follows: dominant leg of the IG, non-dominant leg of the IG, dominant leg of the CG, and non-dominant leg of the CG. The reliability values of the used methods are shown in Table 2.

**Table 3:** Descriptive statistics and two-way ANOVA of maximal strength tests and muscle thickness.

Parameter	Pre-test (m ± SD)	Post-test (m ± SD)	Change (m ± SD)	Time effect	Group effect	Time×group
**Maximal strength**						
IGDL	789 ± 173.6 N	823.8 ± 190.5 N	+4.4% ± 5.4%	*p* = 0.051 *F* = 6.605 $\eta_{p}^{2}$ = 0.046	*p *= 0.142 *F* = 1.864 $\eta_{p}^{2}$ = 0.064	*p* < 0.001 *F* = 6.605 $\eta_{p}^{2}$ = 0.195
IGnDL	695.1 ± 180.1 N	698.2 ± 181.5 N	+0.4% ± 3.9%			
CGDL	816.1 ± 168.0 N	817.8 ± 179.9 N	+0.2% ± 5.5%			
CGnDL	747.8 ± 187.5 N	742.9 ± 189.5 N	−0.7% ± 3.6%			
**Muscle thickness**						
IGDL	154.5 ± 26.3 mm	164.8 ± 27.7 mm	+6.7% ± 7.9%	*p* = 0.001 F = 12.037 $\eta_{p}^{2}$ = 0.132	*p =* 0.398 F = 2.920 $\eta_{p}^{2}$ = 0.037	*p* = 0.039 F = 2.920 $\eta_{p}^{2}$ = 0.100
IGnDL	154.2 ± 26.1 mm	158.0 ± 24.2 mm	+2.5% ± 6.0%			
CGDL	163.4 ± 24.4 mm	165.3 ± 23.2 mm	+1.2% ± 6.8%			
CGnDL	167.6 ± 23.1 mm	168.3 ± 22.3 mm	+0.4% ± 5.1%			

IG, intervention group; CG, control group; DL, dominant leg; nDL, non-dominant leg.

### Maximal strength and muscle thickness

Comparisons of mean values of isometric MSt and MTh in pre- and post-tests for both groups and each leg and the results of ANOVA are presented in Table 3.

The Scheffé test revealed significant increases in mean differences between pre- and post-test values of the stretched leg versus control leg of the IG (*p *= 0.006, *d* = 0.651), stretched leg versus dominant leg of the CG (*p* = 0.031, *d* = 0.55), and stretched leg versus non-dominant leg of the CG (*p* = 0.002, *d* = 0.71). No further significant differences were determined.

MTh *via* sonography Scheffé *post hoc* test showed no significant increases between pre- and post-test values in the stretched leg versus control leg of the IG (*p *= 0.325, *d* = 0.344) or stretched leg versus dominant leg of the CG (*p *= 0.136, *d* = 0.438) nor in the stretched leg versus non-dominant leg of the CG (*p *= 0.066, *d* = 0.505).

### Flexibility

Comparisons of mean values and statistics of ANOVA for flexibility in the measured movement tasks from pre- to post-tests for both groups and each leg are presented in Table 4.

**Table 4:** Descriptive statistics and two-way ANOVA of flexibility.

Parameter	Pre-test (m ± SD)	Post-test (m ± SD)	Change (m ± SD)	Time effect	Group effect	Time×group
**Flexibility of the knee joint**						
IGDL	122.6 ± 8.9°	112.3 ± 9.5°	+8.4% ± 4.2%	*p* < 0.001 *F* = 41.379 $\eta_{p}^{2}$ = 0.320	*p *= 0.398 *F* = 0.997 $\eta_{p}^{2}$ = 0.033	*p* < 0.001 *F* = 26.815 $\eta_{p}^{2}$ = 0.478
IGnDL	120.4 ± 8.8°	120.6 ± 8.4°	−0.2% ± 2.3%			
CGDL	121.8 ± 8.4°	120.7 ± 8.3°	+0.9% ± 2.6%			
CGnDL	120.1 ± 7.4°	119.1 ± 7.0°	+0.8% ± 2.2%			
**Flexibility of hamstrings**						
HIGDL	77.2 ± 10.2°	90.2 ± 11.9°	+16.8% ± 9.7%	*p *< 0.001 *F* = 31.242 $\eta_{p}^{2}$ = 0.262	*p *= 0.679 *F* = 0.507 $\eta_{p}^{2}$ = 0.017	*p* < 0.001 *F* = 30.359 $\eta_{p}^{2}$ = 0.509
IGnDL	79.8 ± 14.6°	79.5 ± 13.7°	−0.4% ± 3.5%			
CGDL	79.0 ± 11.6°	78.9 ± 11.0°	−0.2% ± 5.5%			
CGnDL	80.8 ± 13.7°	80.3 ± 12.6°	−0.6% ± 3.5%			
**Flexibility of the ankle joint**						
IGDL	16.0 ± 2.5 cm	17.5 ± 2.6 cm	+9.4% ± 8.4%	*p* < 0.001 *F* = 9.592 $\eta_{p}^{2}$ = 0.098	*p *< 0.028 *F* = 3.170 $\eta_{p}^{2}$ = 0.098	*p* < 0.001 *F* = 13.183 $\eta_{p}^{2}$ = 0.310
IGnDL	15.1 ± 2.2 cm	15.0 ± 2.3 cm	−0.6% ± 5.4%			
CGDL	16.0 ± 2.3 cm	15.8 ± 2.5 cm	−0.7% ± 7.0%			
CGnDL	14.9 ± 1.9 cm	15.1 ± 2.0 cm	+1.0% ± 4.2%			

IG, intervention group; CG, control group; DL, dominant leg; nDL, non-dominant leg.

The Scheffé test showed significant increases in the stretched leg compared to the control leg within IG in all flexibility tests (Kj: *p* < 0.001, *d* = 1.239; Ham: *p* < 0.001, *d* = 1.247; Aj: *p* < 0.001, *d* = 0.926) as well as compared to the dominant leg of the CG (Kj: *p* < 0.001, *d* = 1.374; Ham: *p* < 0.001, *d* = 1.39; Aj: *p* < 0.001, *d* = 0.844) and the non-dominant leg of the CG (Kj: *p* < 0.001, *d* = 1.216; Ham: *p* < 0.001, *d* = 1.409; Aj: *p* < 0.001, *d* = 0.904). No further significant differences were obtained.

### Muscle stiffness and contraction time

Comparisons of mean values and changes in TMG Dm and Tc in pre- and post-tests for both groups and each leg are presented in Table 5. The intervention did not cause any significant change in these parameters.

**Table 5:** Descriptive statistics and two-way ANOVA of TMG Dm and Tc.

Parameter	Pre-test (m ± SD)	Post-test (m ± SD)	Change (m ± SD)	Time effect	Group effect	Time×group
**Muscle displacement**						
IGDL	8.1 ± 1.7 mm	7.7 ± 1.9 mm	−4.5% ± 17.9%	*p* = 0.316 *F* = 1.016 $\eta_{p}^{2}$ = 0.012	*p *= 0.067 *F* = 1.33 $\eta_{p}^{2}$ = 0.080	*p* = 0.940 *F* = 0.133 $\eta_{p}^{2}$ = 0.005
IGnDL	6.7 ± 1.5 mm	6.6 ± 2.1 mm	−1.3% ± 12.4%			
CGDL	7.6 ± 2.0 mm	7.6 ± 2.4 mm	−0.3% ± 21.6%			
CGnDL	6.9 ± 1.9 mm	6.8 ± 1.8 mm	−0.7% ± 19.7%			
**Contraction time**						
IGDL	29.8 ± 4.6 ms	31.4 ± 4.5 ms	+5.5% ± 9.5%	*p* = 0.119 *F* = 2.478 $\eta_{p}^{2}$ = 0.029	*p *= 0.139 *F* = 1.182 $\eta_{p}^{2}$ = 0.064	*p* = 0.322 *F* = 1.182 $\eta_{p}^{2}$ = 0.041
IGnDL	29.9 ± 4.0 ms	30.3 ± 4.2 ms	+1.3% ± 11.7%			
CGDL	29.5 ± 4.1 ms	30.0 ± 3.9 ms	+1.6% ± 9.6%			
CGnDL	28.1 ± 4.1 ms	27.7 ± 4.1 ms	−1.2% ± 9.3%			

IG, intervention group; CG, control group; DL, dominant leg; nDL, non-dominant leg.

### Creatine kinase

Comparisons of mean values of CK from the pretest to 3 days after the start of the intervention are presented in Table 6. No significant changes were obtained.

**Table 6:** Descriptive statistics and two-way ANOVA of creatine kinase.

Group	Pre (m ± SD)	Post-24 h (m ± SD)	Post-48 h (m ± SD)	Post-72 h (m ± SD)	Time effect	Group effect	Time×group
**Creatine kinase**							
Intervention group	153.1 ± 81.1 U/l	180.8 ± 88.2 U/l	160.5 ± 92.7 U/l	182.8 ± 109.9 U/l	*p* = 0.198 *F* = 1.582 $\eta_{p}^{2}$ = 0.040	*p *= 0.194 *F* = 1.747 $\eta_{p}^{2}$ = 0.044	*p* = 0.290 *F* = 1.265 $\eta_{p}^{2}$ = 0.032
Control group	143.5 ± 74.4 U/l	151.6 ± 66.5 U/l	147.0 ± 78.5 U/l	130.9 ± 69.7 U/l			

## Discussion

The present study aimed to investigate the effects of a self-performed, daily SST for 5 min on leg muscles over 6 weeks on MSt, MTh, and flexibility. Since there is a lack of knowledge on physiological and functional changes following SST, acute changes of serum CK and measurements of contraction properties were included. The results confirm the hypothesis of an increase in MSt and flexibility in the stretched leg compared to the control leg (contralateral and in the control group). However, the results do not confirm the hypotheses concerning an increase in MTh, a decrease in contraction velocity, and a reduction in muscle stiffness. Since CK values are not significantly different, the hypothesis that SST can lead to acute microtraumtization of the muscle is not confirmed. Increases in MSt found in the present study are in line with previous studies (4, 5, 12–14) and could possibly be explained by neuronal or morphological changes due to high mechanical tensile tension. Since an increase in muscle protein synthesis due to stimulated anabolic signaling pathways such as PI3K/Akt/mTOR was reported in animal studies using chronic stretching (26–28), it could be speculated that similar adaptations could occur in humans. Some authors describe a possible stretch-induced stimulation of the PI3K/AkT/mTOR signaling pathway in a muscle through the release of growth factors, such as insulin-like growth factor I and hepatocyte growth factor (28–30). However, the stimulation of anabolic pathways would probably mainly promote hypertrophy, which failed to reach the significance level in this study. Thus, further research seems necessary to investigate the underlying mechanisms of stretch-mediated MSt increases since the contribution of MTh increases to enhanced MSt was limited in this study.

Smith et al. (9) could show a significant increase in CK after static stretching in humans, indicating microtraumatization of the muscles, which may initiate repair mechanisms and stimulate anabolic processes (10, 32), e.g., increase muscle protein synthesis. Even though there seems to be a link between microtraumatization after resistance training—so-called exercise-induced muscle damage and increased hypertrophy (33)—results of the present study were unable to show stretch-induced increases in CK values nor increased MTh. When interpreting increased CK values as a predictor of muscle microtraumatization, a distinction has to be made between statistical evaluation and clinical relevance. There is a wide range of measured CK values in training studies, varying from 20 to 35 to 200–400 U/L at the base level (34, 35) up to 25.000 U/L 1 day after eccentric exercise (36). The mean CK values measured in the present study were 153.1 U/L at the pretest to 180.8 U/L one day after the stretch, while Smith et al. (9) measured a mean CK of 84.5 U/L at the pretest and 126.7 U/L one day after static stretching, which was the highest measured CK value. However, the CK values of Smith et al. (9) are still within the range of an average base level of CK and can be considered within a normal non-muscle-damaged physiological range of variation. exercise-induced changes in CK have their peak 3 days after training (37). Therefore, in the present study, CK was measured before intervention, 1, 2 and 3 days after the start of the intervention. However, all CK values are within an average base level of CK (34) and can be classified as a non-muscle-damaged physiological range of variation. Therefore, it can be assumed that 4 × 5 min of daily SST on the first 3 days of the intervention period did not lead to a clinically relevant increase in CK values, indicating no acute microtraumatization response of muscle tissue.

Goldspink and Harridge (10) describes a link between cross-sectional area of the muscle and the force production potential of the muscle. However, muscle hypertrophy can also occur without microtraumatization of the muscles (38). Previous studies observed significant MTh increases following SST; a hypertrophy effect could possibly explain the MSt increases (4, 8, 39). Yahata et al. (14) examined the effect of SST on the gastrocnemius using 6 × 5 min per session on 2 days per week for 5 weeks and observed a significant increase in MSt but not in MTh, which was similar to the present study. The present results suggest that too low stretch intensity could be responsible for no changes in MTh. In self-performed SST, stretch intensity is limited by the subjective level of tolerated pain. On a VAS of 1–10, which describes the maximum stretch pain that can be tolerated, participants in the present study were instructed to perform the exercises to the maximum tolerated stretch pain. However, since the VAS is subjective and the perception of pain varies greatly from person to person (40), it can be speculated that the stretch intensity may not be sufficient. Lim and Park pointed out no correlation between passive peak torque and stretch pain. In contrast to the present study, Warneke et al. (4) observed a +15.3% increase in MTh of the calf muscle. As participants stretched the calf muscles for 7 h every week, the training volume was more extensive than the present study. A study by Simpson et al. (8) used an external stretching device and observed a significant increase in MTh. However, they performed a stretch volume of 900 s compared to the 2100 s per muscle used in the current study. It can be hypothesized that passive SST with an external stretching device leads to greater MSt and MTh increases than self-performed SST.

Since this is the first study examining muscle stiffness and contraction velocity by tensiomyography following 6-week SST, there is limited comparability to the results of other studies. In the present study, no stretch-induced increase in Tc was measured, indicating no decrease in contraction velocity. Since in animal models, Alway (1) used a stretching duration of 24 h per day, compared to 5 min per day in the present study, an insufficient stretch time could be assumed to induce a change in contraction velocity.

Literature provides different results about changes in muscle stiffness after several weeks of long-term SST. While there is evidence that stiffness of the muscle–tendon complex decreases after several weeks of SST (15, 16, 41), other studies did not find any changes in stiffness (17, 18). There are different methods of examining muscle stiffness, like dynamometer and sonography (16–18) or shear wave elastography (42). In the present study, muscle stiffness was determined *via* TMG with an involuntary contraction by measuring muscle belly displacement. Nakamura et al. (41) measured a significant decrease in muscle stiffness following 6 weeks of SST on 3 days per week. The participants performed stretching using a stretch board and reached maximal stretch pain. In the present study, participants stretched without any stretch device. It could be hypothesized that the intensity of the stretch was also not sufficient to induce changes in muscle stiffness.

As expected, in all measured flexibility tests after 6 weeks, stretch training resulted in an improvement of flexibility. Weppler and Magnusson (43) suggested considering two underlying mechanisms: First, an increase in pain tolerance due to a change in the sensitivity of peripheral nociceptors, which is supported by other Authors (44, 45), and second, structural adaptations of the muscle, e.g., decreased muscle stiffness or increased muscle fascicle length (43). The explanation for an increase in pain tolerance could be less important for flexibility tests that do not reach maximum pain tolerance. The measured flexibility of the knee joint in the Thomas test in the present study was not limited by maximum tolerated pain but rather by gravitational force. Since the rectus femoris muscle–tendon complex is connected to the patella and tibialis, the only force that bends the lower leg at the knee joint to achieve a change in flexibility is the gravitational force. Gravitational force can be assumed to not change, and muscle stiffness in the rectus femoris measured *via* TMG remains also unchanged from pre- to post-tests (Dm: *p* = 0.94). Animal studies show an increased number of sarcomeres in series in response to stretching (28). A fascicle length increase can be hypothesized due to adding sarcomeres in series. However, there is very limited evidence for longitudinal hypertrophy in humans. Since no structural or neuromuscular testing was performed to explain increased flexibility, explanatory approaches for increased flexibility remain hypothetical. Further research is necessary with flexibility tests measuring a change in flexibility without limitation by maximum pain.

In conclusion, self-performed SST for 5 min per muscle group over 6 weeks led to significant increases in MSt and flexibility. Since acute CK did not increase, microtraumatization of the muscle due to SST can be considered unlikely. Similarly, no significant increase in MTh compared to the non-stretched leg could be detected. Therefore, morphological adaptations cannot explain the increased MSt in the current study.

## Outlook

More research is required to explain increases in MSt and flexibility. Future studies should include comparisons of passive SST with an external stretch device and self-performed SST since the literature shows greater MSt and MTh increases when using an external stretch device. Since changes in muscle stiffness were found in human studies, and contraction velocity was found in animal studies using longer stretch durations, further studies should include longer stretch durations to investigate physiological changes of SST. If stretch time is greater than 5 min, oxygen saturation decreases to 36% (hypoxia condition) (46). Therefore, a long-term effect on contraction velocity due to hypoxic conditions could be hypothesized if stretching is held for more than 5 min, especially using an external stretch device.

## Limitations

It should be mentioned that sonography for MTh determination has some weaknesses in terms of reliability, such as how much probe pressure is applied to measure tissue or water retention. Especially in longitudinal studies, the reliability of ultrasound images should be considered critically (47). To counteract this problem as best as possible, we took three images of one leg and plotted three distances each. This resulted in nine measured values for MTh per test, from which a mean value was calculated. The tester was always the same. Furthermore, muscle architecture changes, for instance, fascicle length and pennation angle, were not measured in the present study, which limited the interpretation of MTh. Therefore, further studies should implement measuring the fascicle length and pennation angle in combination with the evaluation of MTh. Moreover, the participants in the present study were not randomized because it was difficult to find subjects willing to perform intensive stretch training daily for 6 weeks. In addition, TMG as a non-invasive method to measure contraction properties due to surface electrodes should be viewed critically when used in longitudinal studies. Electrode placement, the amount of water in the tissue, and the subcutaneous fat could influence the measurement (24, 25). Muscle stiffness is also not measured directly with TMG. The measurement of the amount of muscle displacement using TMG can only provide indirect information on muscle stiffness during an involuntary contraction. In further studies, it is recommended to measure passive muscle stiffness too by using, e.g., shear wave elastography. Since CK was measured 3 days following SST, there are no long-term data on potential muscle microtraumatization.

## Data Availability

The raw data supporting the conclusions of this article will be made available by the authors, without undue reservation.
